# Quality of Documentation as a Surrogate Marker for Awareness and Training Effectiveness of PHTLS-Courses. Part of the Prospective Longitudinal Mixed-Methods EPPTC-Trial

**DOI:** 10.1371/journal.pone.0170004

**Published:** 2017-01-20

**Authors:** David Häske, Stefan K. Beckers, Marzellus Hofmann, Rolf Lefering, Bernhard Gliwitzky, Christoph C. Wölfl, Paul Grützner, Ulrich Stöckle, Marc Dieroff, Matthias Münzberg

**Affiliations:** 1 Faculty of Medicine, Eberhard Karls University Tübingen, Tuebingen, Germany; 2 Department of Anesthesiology, University Hospital RWTH, Aachen, Aachen, Germany; 3 Emergency Medical Service, Fire Department, City of Aachen, Aachen, Germany; 4 Faculty of Health, University of Witten/Herdecke, Witten, Germany; 5 Institute for Research in Operative Medicine, University of Witten/Herdecke, Cologne, Germany; 6 Megamed Emergency Management GbR, Annweiler, Germany; 7 Department of Orthopedics, Trauma Surgery and Sports Traumatology, Hospital Hetzelstift, Neustadt, Germany; 8 Department of Trauma and Orthopedic Surgery, BG Trauma Centre Ludwigshafen, Ludwigshafen, Germany; 9 Department of Traumatology and Reconstructive Surgery, BG Hospital Tuebingen, Tuebingen, Germany; 10 Fire Service, Emergency Preparedness and Crisis Management, City of Wiesbaden, Wiesbaden, Germany; Yokohama City University, JAPAN

## Abstract

**Objective:**

Care for severely injured patients requires multidisciplinary teamwork. A decrease in the number of accident victims ultimately affects the routine and skills. PHTLS (“Pre-Hospital Trauma Life Support”) courses are established two-day courses for medical and non-medical rescue service personnel, aimed at improving the pre-hospital care of trauma patients worldwide. The study aims the examination of the quality of documentation before and after PHTLS courses as a surrogate endpoint of training effectiveness and awareness.

**Methods:**

This was a prospective pre-post intervention trial and was part of the mixed-method longitudinal EPPTC (Effect of Paramedic Training on Pre-Hospital Trauma Care) study, evaluating subjective and objective changes among participants and real patient care, as a result of PHTLS courses. The courses provide an overview of the SAMPLE approach for interrogation of anamnestic information, which is believed to be responsible for patient safety as relevant, among others, “Allergies,” “Medication,” and “Patient History” (AMP). The focus of the course is not the documentation.

**Results:**

In total, 320 protocols were analyzed before and after the training. The PHTLS course led to a significant increase (p < 0.001) in the “AMP” information in the documentation. The subgroups analysis of “allergies” (+47.2%), “drugs” (+38.1%), and “medical history” (+27.8%) before and after the PHTLS course showed a significant increase in the information content.

**Conclusion:**

In summary, we showed that PHTLS training improves documentation quality, which we used as a surrogate endpoint for learning effectiveness and awareness. In this regard, we demonstrated that participants use certain parts of training in real life, thereby suggesting that the learning methods of PHTLS training are effective. These results, however, do not indicate whether patient care has changed.

## Introduction

Accidents have the most common cause of death since the middle ages [1]. In Germany, 4.7 deaths per 100,000 inhabitants have been reported, and in the United States (US), 11.4 per 100,000 inhabitants [2]. The peak age is between 18 to 25 years [3]. These young employed people particularly benefit from rapid rehabilitation after an accident. In Germany alone, the economic costs of traffic accidents amount to 31 billion euros [4].

Fortunately, there is a continuous decrease in the number of accidents [5,6]. The proportion of injured patients, based on the sum of all emergency patients in Germany, is approximately 10% [7]. The decreasing number of accident-related emergency calls destabilizes the routine [8]. This underlines the importance of effective training in emergency medicine.

In the 1970s, the treatment of trauma patients in the emergency room became increasingly standardized, following the introduction of Advanced Trauma Life Support (ATLS), which provided a new structure in the care for severely injured patients [9]. The associated pre-hospital care equivalent to ATLS is the Pre-Hospital Trauma Life Support (PHTLS) concept. There are also other training concepts, but PHTLS is an established concept in 66 countries around the world. Induction into PHTLS is delivered through two-day courses for medical providers, with the aim of improving the pre-hospital care of trauma patients.

In the late 1990s, Ali et al. showed that, in less developed emergency medical services (EMS) systems, PHTLS improves skills and procedures [10] and leads to a significant reduction of mortality [11,12]. However, these results cannot be transferred to current and developed EMS systems. A Scandinavian observational study showed that PHTLS training is associated with a small reduction in mortality (the mortality risk was 4.7% (36/763) without PHTLS training and 4.5% (94/2067) with PHTLS training) [13]. In a subgroup analysis of motor-vehicle traffic injuries, no reduction of mortality was observed [14]. It is questionable if the end-point “mortality” is sufficient to evaluate the effects of PHTLS in modern emergency care.

### Importance

In EMS-district Wiesbaden (Germany), a previously used training concept has been revised due to the lack of learning success and employee satisfaction. At the instigation of the medical director, PHTLS courses were mandatorily established for all paramedics in EMS-district Wiesbaden [15].

Against the background of a large EMS district introducing PHTLS as standard training, with mortality as an endpoint having demonstrated little advantage of PHTLS in previous studies, the goal of this project was to analyze subjective and objective changes among participants and in real patient care, as a result of PHTLS courses.

### Goals of this investigation

The aim of the study was to investigate documentation quality before and after PHTLS courses, as a surrogate marker of training effectiveness and awareness.

## Materials and Methods

### Study design

This was a prospective pre-post intervention trial and was part of the mixed-methods longitudinal EPPTC (Effect of Paramedic Training on Pre-Hospital Trauma Care) study, evaluating the subjective and objective changes in participants and real patient care, as a result of PHTLS courses. The complete study is described in the previously published study protocol [16].

### Research ethics approval

The Ethics Committee of the Medical Faculty of the Eberhard Karls University of Tuebingen and the University Hospital approved the study proposal, number 197/ 2013BO2, on May 24, 2013. The study is registered in the German Clinical Trials Register as ID, DRKS00004713. Data collection, coding, routing, and analysis were coordinated by a data protection officer at the University of Tuebingen and the University Hospital of Tuebingen. In another part of the project, participants were surveyed through a questionnaire, after providing written consent. For this part of the study, no personal information from participants or patients was collected.

### Study setting and selection of participants

The study was performed in the EMS in Wiesbaden (Germany). The operational district in Wiesbaden has five commissioned EMS agencies (four charities, one private provider). The EMS in Wiesbaden had 375 paramedics and served 462,098 inhabitants during the study period of 2013/2014.

In the context of various difficulties and problems, the controlling authority committed all paramedics to attend PHTLS courses, so as to establish uniform structures and principles [15].

### Intervention

PHTLS courses are well-established worldwide, comprising two-day courses for paramedics and emergency physicians, with the aim being to improve pre-hospital care for trauma patients. PHTLS courses are characterized by a large variety of teaching methods (e.g., lectures, practical case studies, and skills training), with a low instructor—participant ratio (1:4), many practical activities, and continuous interaction. In addition to various skills, the priority-based structure, ABCDE (Airway, Breathing, Circulation, Disability and Exposure), is taught and practiced in scenario-based training sessions. The teaching of PHTLS conforms with a high conformity to the key recommendations of the German “Guideline on Treatment of Patients with Severe and Multiple Injuries” [17].

“ABCDE” represents the core strategy and has the highest attention during the course. With regard to the awareness and transfer of teaching content to the real working world, a surrogate parameter, which was not so focused in the courses, seemed more appropriate. As part of the so-called “secondary assessment,” PHTLS courses, as well as other courses like ATLS and AMLS, use “SAMPLE” as a mnemonic tool [18]. The letters “AMP” are an abbreviation for allergies, medication and patient history (pre-existing illness). The analysis reviewed these items in the standardized EMS protocols from actual field operations performed by paramedics. The focus of the lesson was not set on the operation documentation. The decision to focus on “AMP” has different reasons. The first is its importance for patient safety [19]. The other reason came up during the initial data analysis, which showed that data processing of “L” and “E” was bugged. To retain data quality, just “AMP” was included.

The state of Hessen used so-called report categories (“RMZ”), in which medical indications (e.g. combustion, hyperventilation) were reported. The main groups are shown in Table 1. To indicate the patient’s condition, an emergency severity score (“RMC”) and timestamps were used (Table 2). The minimum number of points assigned as the RMC is six; the maximum number of points is 42. Deviations from the physiological condition of the patient are indicated by RMC > 6. There was no validation of correct assessment or use by the paramedics or emergency physicians.

**Table 1:** Main groups and number of report categories.

Main groups	Number of report categories
Reanimation	2
Surgical emergencies	46
Internal emergencies	60
Neurological emergencies	18
Pediatric emergencies	8
Gynecological emergencies	12
Other emergencies	22

**Table 2:** Emergency severity score (RMC): Emergency severity is mapped through a six-digit number (RMC). Each digit reflects the patient's condition (at first contact) in relation to the characteristics of consciousness, respiration, circulation, injury, neurological condition, and pain again. The minimum value of "1" means "inconspicuous," in reference to each characteristic; the maximum value of "5" refers to an extremely severe degree of the relevant impairment.

Classification	Consciousness	Respiration	Circulation	Injury	Neurology	Pain
1	Normal	Without	Without	None	Without	None
2	Somnolent	Slightly abnormal	Slightly abnormal	Slight	Previously known disorder	Slight 3
3	Potential loss of consciousness	Potentially	Threatening severe disorder	Conceivable	Threatening disorder	Moderate 4–6
4	Comatose I–III	Severe disorder	Severe disorder	Severe	Acute disorder	Strong7–9
5	Comatose IV	Apnea	Pulseless	Multi-system trauma	Progressive Disorder	Extreme 10

### Data collection and processing

The EMS protocols were required in the context of each EMS field operation and archived at the EMS agencies, in concordance with the local EMS regulations and supervisory authority. We collected the first protocols the year before and after the courses; the inclusion criteria were all operations without emergency physicians and RMC > 6. Because the highest impact is given when participants use SAMPLE for all emergency patients, we included all kind of emergencies, not only trauma patients.

### Outcome measures

The primary outcome measure was a change in the quality of documentation, as a surrogate endpoint for training effectiveness and awareness. Secondary outcome measures were subgroup analyses for differences between the documentation items in the main diagnosis groups (based on the report digits) and the group of patients in severe conditions (based on the emergency severity score).

### Primary data analysis

The sample size of 320 cases per group (pre; post) was based on a detectable difference of 15, with a power of 80%. Type I error was probably 0.05.

The protocols were selected for participating EMS agencies, proportional to their level of participation in the total operations protocols.

The documentation items were offset by one point for each completed item. With a total of three available documentation items (AMP), 0–3 points per protocol were possible, the total of which was obtained for the pre- and post- groups. In each group, a minimum of 0 points and a maximum of 960 points (3 × 320 protocols) were possible.

### Statistical analysis

Formal pre- and post-intervention statistical evaluation was performed with Mann-Whitney’s *U* test for continuous variables and Fisher's Exact Test for categorical variables. A two-tailed test, with p < 0.05 was considered statistically significant. All data were analyzed using the statistical software, SPSS (Version 23.0, IBM Inc., Armonk, NY, USA). For continuous variables, data are shown as mean ± standard deviation. For categorical variables, percentages are presented.

## Results

**Fig 1:**
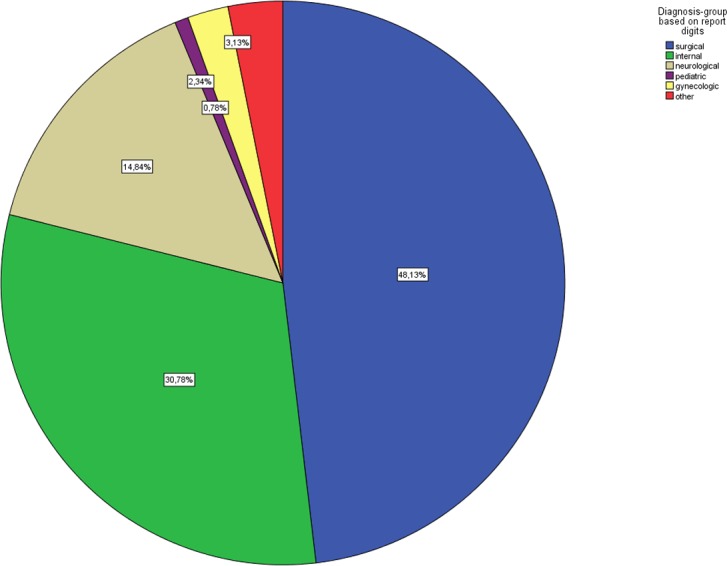
Overall range indications of the selected emergency operations.

In total, 640 protocols (320 for each year) were included in the study. The two most common indications were surgical (n = 308, 48.1%) and internal (n = 197, 30.8%) emergencies (Fig 1).

**Fig 2:**
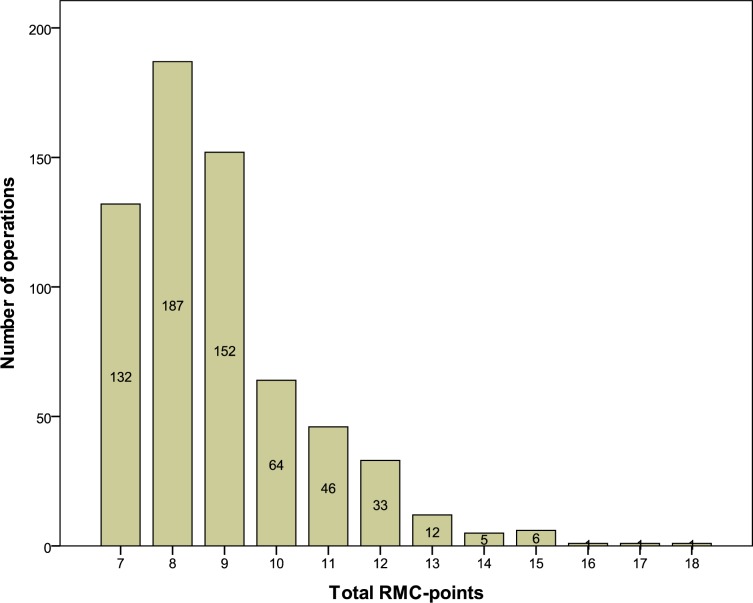
Total number of RMC-points and number of operations.

The mean (with standard deviation) of the RMC (emergency severity score) was 8±2 in both years; the median prior to training was 9, and 8, after training. Fig 2 shows an overall right-skewed distribution, with 1.42.

**Fig 3:**
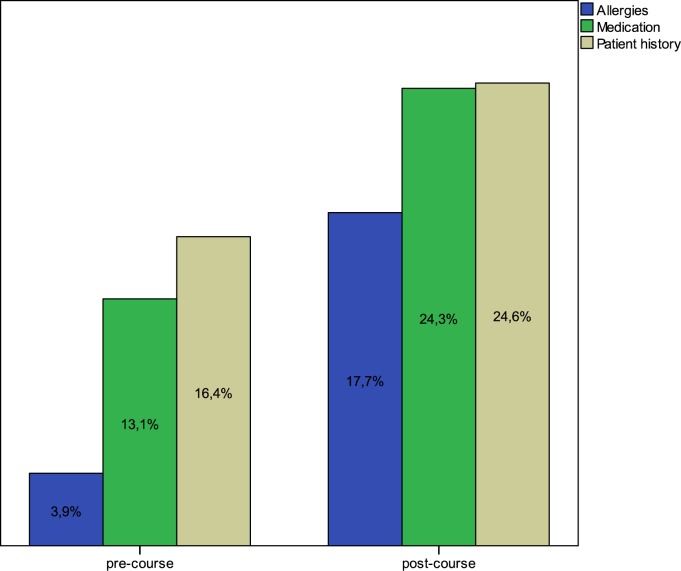
Total “AMP” points were 364 points in the pre-course group and 726 points in the post-course group (p < 0.001).

The totals of the documentation items (AMP) were n = 364 points in the pre-course group and n = 726 points in the post-course group (p < 0.001), which made up an overall increase of 37.7% (Fig 3).

The shifts in the subgroups’ allergy, medication, and patient history were also significant (p < 0.001), as shown in Table 3.

**Table 3:** A comparison of allergy, medication, and patient history before and after the PHTLS course.

	Pre-course	Post-course	Difference	p-value
Allergy	42	193	+47.2%	<0.001
Medication	143	265	+38.1%	<0.001
Patient history	179	268	+27.8%	<0.001

**Fig 4:**
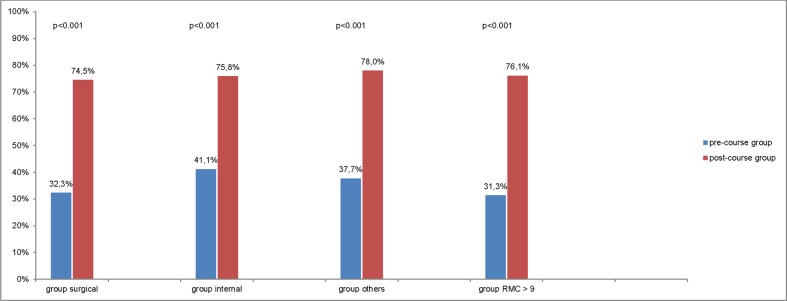
Subgroup analysis of the indication groups, including surgical, internal, and all other indications, as well as operations with RMC- > 9, showed an increase in documentation values (p < 0.001).

According to the indications of operations, a subgroup analysis of surgical and internal indications, and all the other indications in one group, as well as a different point of view on operations with RMC > 9, also showed a significant increase in documentation values (Fig 4).

## Discussion

In this trial, we used the quality of documentation as a surrogate endpoint for the training effectiveness of PHTLS courses in 640 operations protocols. Our score, related to the three items, allergy, medication, and patient history, showed a significant increase of 37.7% after the courses. Based on the emergency severity score or “RMC,” Fig 2 shows that most of the patients were not severely injured or sick. The subgroup analysis of the operation protocols of most severely injured or sick patients, respectively, with RMC > 9, confirmed the increase by a significant +44.8%. The individual documentation items also increased by 27.8% for *patient history*, 38.1% for *medication*, and a notable 47.2% for *allergy*.

Considering the subgroups’ surgical and internal indications, it seems that the largest increase among both groups was the 42.1% observed in the surgical group. Improvement was also observed in the internal group (34.7%). The baseline value of the fulfilled "AMP"-information was 41.1% in the internal group and 32.3% in the trauma group. Based on the relative proportions of the indications, it is arguable as to whether trauma training is responsible for the larger increase in the trauma group or if for example discussion about new anticoagulation [20–22] increased documentation.

RMC (emergency severity score) was 8±2 in both years, this shows that patients in our investigation were not seriously ill or injured. This maybe represents a typical patient collective for a large city. The extent to which our results can be transferred to the stressful emergency care of seriously injured patients is unclear.

Overall, the quality of documentation increased significantly, leading to the notion that PHTLS courses influence participants, with remarkable effects on real patient care and documentation.

Despite the increase in the quality of documentation, documentation still needs to be improved in 24.4% of the cases, even after the courses.

The loss of relevant medical information (e.g., during handovers) is a known problem that concerns not only documentation, but also verbal handovers [23]. This particularly affects the collection of medical history and physical examination results [24]. A video-based error analysis, as conducted by Bergrath et al., of documentation by physicians, following simulation, showed that 20% of the information was missing and 22% of the documented information was incorrect [25].

It has been shown that training or tutorials on documentation improve the quality of documentation [26–28] by 12.5% to 51%. In contrast to cited studies, in which improvement in documentation was considered an endpoint, in our trial, secondary assessment with queries on allergies, medication, and patient history, based on the learned SAMPLE schema, was only a secondary aspect, preceded by use of documentation. Nonetheless, our investigation showed an overall increase in documentation quality by 37.7%, which we think is remarkable.

It could already be demonstrated that the use of protocols, which have integrated memory aids, significantly reduce documentation errors [29]. In the present study, however, the operation protocols had memory aids, such that analyzed items had to be documented in a free-text field.

The results also indicate that, as already known [30], obvious educational interventions may generally have an impact on the system or students. This is due to not only improvement in the documentation for trauma and surgical patients, respectively, but for all other operations, too. Despite the internal medicine group’s initial documentation, which was already better, at 41%, compared to the surgical group, at 32%, an increase on 75.8% was also noted.

This result may also be explained by increased awareness regarding the three items “AMP”. Such increased awareness can be seen partly through simple educational interventions aimed at the public, in relation to stroke detection [31].

For healthcare professionals, awareness is one of the main elements of non-technical skills and a characteristic of high-performing teams [32]. The results of the present study show that awareness can be instilled via training, without much effort. Often, the amount of information in a given (emergency) situation causes an information overload, because the information is not adequately prioritized and categorized [33]. The taught SAMPLE scheme seems to bring some order, in this regard.

This trial, like many others in medical and complementary disciplines, uses surrogate endpoints, which have no value of their own [34–39]. They simply constitute indirect evidence for training effectiveness in this study. Therefore we decided not to use the ABCDE, because this was highlighted in the PHTLS trainings. Also, documentation of ABCDE is quite more difficult. In the care for less injured patients with no ABCDE problem, the need of documentation of the absence of a problem is forgotten easily. For example a patient with extremity injury without analgesia has not a worse treatment, without documentation of the uncompromised airway and breathing. The need of documentation of the SAMPLE information is more obvious—even if negative, for instance “no allergies” or “no medication”. On the other hand “SAMPLE” was just slightly taught in one lesson, but is important for every patient. So the transfer of this into real patient documentation shows the big effects of one training on awareness and the learning process.

An alternative validation could have been the use of a written exam to evaluate training effectiveness, similar to Ali et al., in 1998, who showed good learning success among PHTLS-course students [40], using a pre-/post-test. However, the aim of our study was to investigate changes in real patient care, as a result of the courses, which would not have been possible with a written exam. This led to the decision to use the documentation quality of real emergency operations as surrogate endpoints for training effectiveness.

The study participants were informed about the study and consented to scientific evaluation. Therefore, a Hawthorne effect [41] could be assumed and was critically discussed. As it had not been announced that the documentation would be evaluated, in our opinion, the Hawthorne effect is not applicable, in this instance.

Due to the high volume of EMS operations, the predetermined sample size of operation protocols was reached within a short period of time, even though the same month was chosen each year to collect protocols.

A follow-up for this part of the trial could not be initiated because subsequent training could falsify the result. Therefore, the continuous quality of documentation, variance thereof, and long-term training effectiveness cannot be verified. These effects could perhaps be improved through refresher training, which was not evaluated in the present study.

## Conclusion

In summary, we showed that PHTLS training improves the quality of documentation, which we used as a surrogate endpoint for learning effectiveness and awareness. In this regard, we demonstrated that participants use certain parts of training in real life, thereby suggesting that the learning methods of PHTLS training are effective. However, these results do not indicate as to whether patient care has changed.
